# Kartagener’s syndrome and psychotic disorder : a case report

**DOI:** 10.1192/j.eurpsy.2026.11087

**Published:** 2026-07-31

**Authors:** I. Ilahi, S. Belkaid, A. Barkati, M. Othmani, H. Maktouf, A. Ben Hamadi, L. Mnif

**Affiliations:** Psychiatry F, Razi Hospital, Manouba, Tunisia

## Abstract

**Introduction:**

Kartagener’s syndrome, is a rare congenital malformation characterised by a triad : situs inversus, chronic sinusitis and bronchiectasis, and has rarely been associated with psychiatric manifestations.

While respiratory and otolaryngologic complications are well documented, little is known about potential links between primary ciliary dyskinesia and neuropsychiatric disorders, including schizophrenia.

**Objectives:**

We report the case of a 21 year old male with no prior history of psychiatric disorders who presented to the emergency room with acute psychomotor agitation and incoherent speech .

**Methods:**

Psychiatric assessment confirmed a first psychotic episode consistent with schizophrenia.

Clinical and radiological evaluation revealed situs incersus and bronchiectasis leading to the diagnosis of kartagener’s syndrome. No prior family psychiatric history or substance abuse was identified .

**Results:**

To our knowledge, this is among the very few reported cases of comorbidity between Kartagener syndrome and schizophrenia. Possible explanatory pathways include shared genetic susceptibilities, immune-inflammatory dysregulation, or neurodevelopmental effects linked to ciliary dysfunction. This case highlights the need to explore whether primary ciliary dyskinesia may predispose to psychiatric vulnerability, particularly psychotic disorders.

**Conclusions:**

This report emphasizes the importance of considering rare somatic comorbidities such as Kartagener syndrome in the evaluation of first-episode psychosis. Further studies are warranted to investigate potential pathophysiological overlaps between ciliary dysfunction and schizophrenia.

**Disclosure of Interest:**

None Declared

